# Structure and Properties of La_2_O_3_-TiO_2_ Nanocomposite Films for Biomedical Applications

**DOI:** 10.1155/2011/853048

**Published:** 2011-11-22

**Authors:** Lin Zhang, Zhi-Hua Sun, Feng-Mei Yu, Hong-Bin Chen

**Affiliations:** ^1^Information College, ZhongKai University of Agriculture and Engineering, Guangzhou 510225, China; ^2^Department of Endocrinology, Guangzhou Panyu Central Hospital, Guangzhou 511400, China

## Abstract

The hemocompatibility of La_2_O_3_-doped TiO_2_ films with different concentration prepared by radio frequency (RF) sputtering was studied. The microstructures and blood compatibility of TiO_2_ films were investigated by scan electron microscopy (SEM), X-ray photoelectron spectroscopy (XPS), and UV-visible optical absorption spectroscopy, respectively. With the increasing of the La_2_O_3_ concentrations, the TiO_2_ films become smooth, and the grain size becomes smaller. Meanwhile, the band gap of the samples increases from 2.85 to 3.3 eV with increasing of the La_2_O_3_ content in TiO_2_ films from 0 to 3.64%. La_2_O_3_-doped TiO_2_ films exhibit n-type semiconductor properties due to the existence of Ti^2+^ and Ti^3+^. The mechanism of hemocompatibility of TiO_2_ film doped with La_2_O_3_ was analyzed and discussed.

## 1. Introduction

With the advancement of organ transplantation, biocompatibility, particularly blood compatibility, becomes the most important property required for biomedical materials. It is desired to develop new biomaterials with good physical, mechanical properties and hemocompatibility. Recent studies have shown that TiO_2_ films are suitable as surface coatings on biomedical applications due to its good hemocompatibility [1, 2], and researches concerning biomedical aspects are widely increasing [3–5]. The emphases of blood compatible materials research are divided into two aspects, one is the surface properties, and the other is the band and electron structure of the biomedical materials.

It is believed that the first step after blood contacting with the biomaterial is adsorption of plasma protein, which will determine the anticoagulation property of the biomaterial. In our previous paper [6], we studied the surface properties of La_2_O_3_-doped TiO_2_ films and investigated the interaction between the material surface and plasma proteins. In this paper, we do the farther work and try to achieve deeper understanding of the mechanisms which are involved in blood-biomaterial interaction by investigating the influence of various La_2_O_3_ concentrations on the electronic structure and hemocompatibility of TiO_2_ nanocomposite films.

## 2. Materials and Methods

### 2.1. Preparation of Thin Films

The La_2_O_3_-doped TiO_2_ films were prepared by the radio frequency (13.56 MHz) magnetron sputtering technique. The n-type Si (100) and quartz were used as the substrates. The targets were mechanically mixed by using TiO_2_ powder (in purity 99.9%) and La_2_O_3_ powder (in purity 99.5%) with the La_2_O_3_ molar concentration of 0%, 1%, 2%, and 3%, respectively. The substrates were ultrasonically cleaned in acetone and then were mounted on the substrate holder. After being evacuated to a base pressure of 3 × 10^−3^ Pa, the working chamber was filled with Ar (99.99% purity) and the Ar gas flow was kept constant at 12 SCCM during the deposition process. The samples were prepared in room temperature with a typical work pressure of 4 Pa. The RF power of 200 W was applied in the sputtering process. The samples S1, S2, S3, and S4 are prepared using the targets with a doped La_2_O_3_ molar content of 0%, 1%, 2%, and 3%, respectively. 

### 2.2. Characterization of Thin Films

Scanning electron microscopy (SEM, QUANTA 400F) was employed to characterize the surface topography of the TiO_2_ nanocomposite films. The compositions were analyzed by X-ray photoelectron spectroscopy (XPS, ESCALAB 250, UK). The XPS spectra were recorded using Mg K_*α*_ (1253.6 eV) X-ray source. The binding energy of the Au 4f_7/2_ core level electron is taken to be 84.0 eV for energy calibration. Spectra were recorded with 20 eV pass energy for the survey scan and with 10 eV pass energy for the La 3d and Ti 2p regions. High resolution XPS conditions have been fixed: “fixed analyser transmission” analysis mode, a 7 × 20 mm entrance slit and 150 W electron beam power. A takeoff angle of 90° from the surface was employed. The spectra were fitted using the Casa XPS v.2.3.13 Software (Casa software Ltd., UK) and by applying a Gaussian/Lorentzian ratio, G/L equal to 70/30. Before measurement, Ar ion etching was performed 8 minutes with an etching rate of 0.025 nm/s in order to eliminate atmosphere contaminants. A double-beam UV-visible spectrometer was used to investigate the optical absorption of the samples. The absorption spectra (in the wavelength range of 200–700 nm) were obtained by using a bare quartz substrate to eliminate the substrate contribution.

## 3. Results and Discussion

### 3.1. Structure Properties

**Figure 1:**
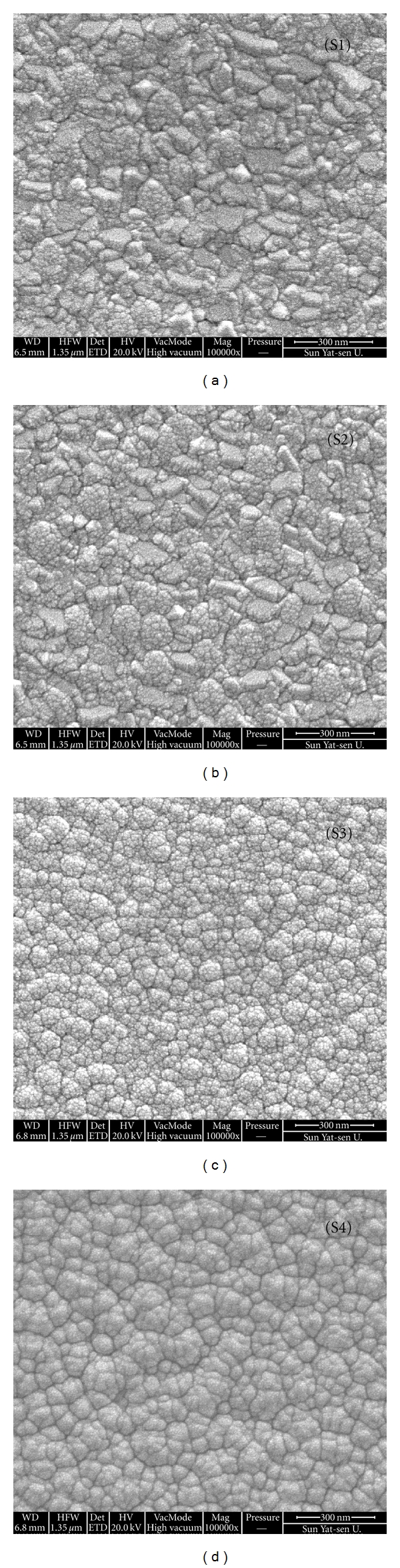
Typical SEM images of the surface images of TiO_2_ thin films doped with La_2_O_3_ with various concentrations (a) S1; (b) S2; (c) S3; and (d) S4.

**Figure 2:**
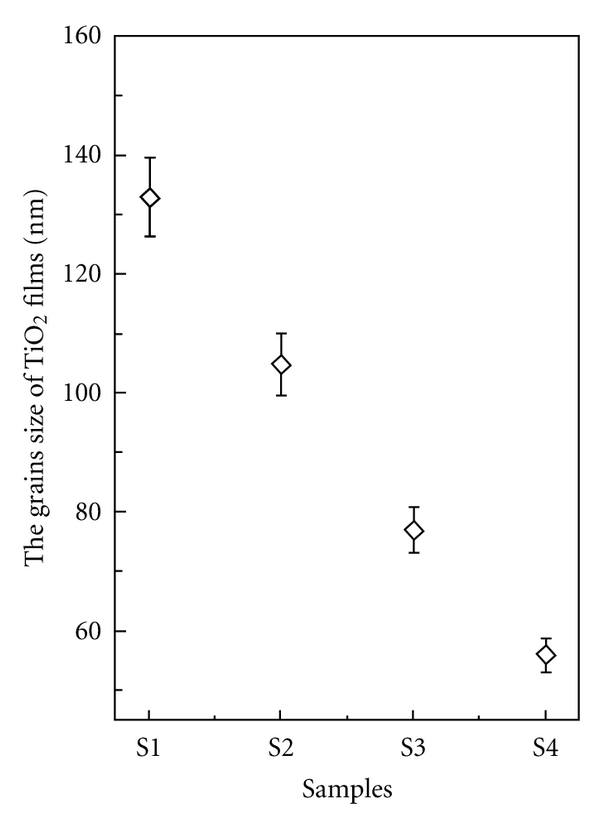
The distribution of grains dimensions.

The SEM photographs of TiO_2_ nanocomposite films are shown in Figure 1. The surface morphology of the films becomes smooth with increasing of the La_2_O_3_ content in TiO_2_ films, and the grains are uniform.  Figure 2 shows the grains size of TiO_2_ films decreases from 133 nm to 56 nm. These micrographs, in combination with results derived by XRD, showed in our previous paper [6], suggest that La dopant not only greatly promotes the phase transfer from anatase to rutile and enhances the crystal phase of TiO_2_ with preferential growth in the direction of (110), but also can refine grain size of TiO_2_.

### 3.2. XPS Characterization

**Figure 3:**
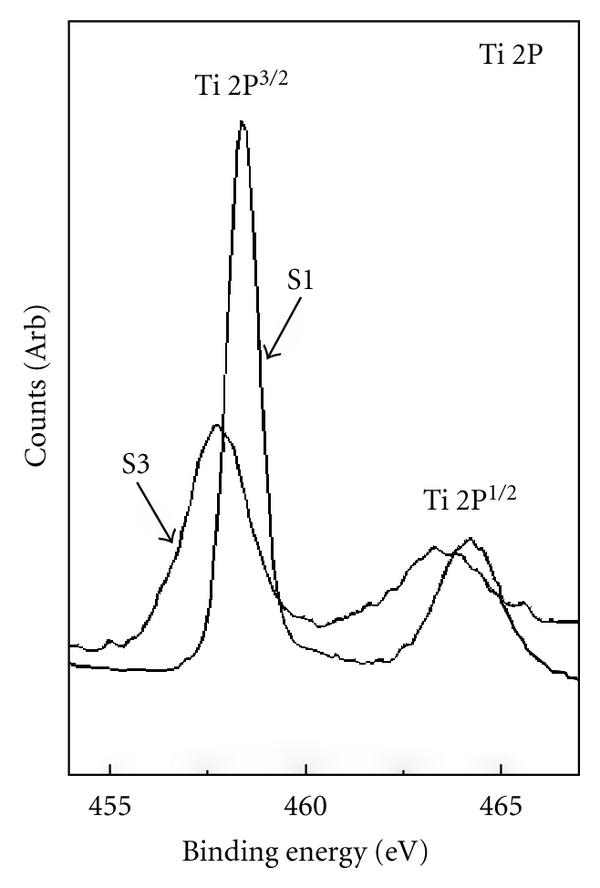
XPS spectrum of the Ti 2p region for the surface of S1 and S3.

The La 3d spectrum of TiO_2_ nanocomposite films, showed in our previous paper [6], indicated that the La exists as La_2_O_3_ in TiO_2_ films. All La_2_O_3_-doped TiO_2_ films show similar results. As a comparison with undoped sample, the high-resolution XPS analysis of the Ti 2p region obtained on the surface of S1 and S3 was shown in Figure 3. Two pronounced features are observed at binding energies near 458.4 eV and 464.3 eV, corresponding to the Ti 2p_3/2_ and Ti 2p_1/2_ states, respectively. The two peaks of S1 are symmetrical, which indicates that Ti exists as Ti^4+^ in TiO_2_ films. It is obvious that doping La_2_O_3_ not only leads to the positions of two peaks of S3 shift towards lower binding energies, but also impels a broad shoulder of the two peaks. The ratio of the area of the two peaks A(Ti 2p_1/2_)/A(Ti 2p_3/2_) is equal to 0.7, and the splitting of the doublet Δ*E*_*b*_ = *E*_*b*_(Ti  2p_1/2_) − *E*_*b*_(Ti  2p_3/2_) is 5.9 eV, which indicates that the doublet was mainly assigned to Ti^4+^ and the minor contributions of Ti^2+^ and Ti^3+^ should be taken into account as follows: (1) Ti^2+^ was from Ti_2_O_3_ species, with the binding energies locating at *E*_*b*_ (Ti 2p_3/2_) = 457.50 eV and *E*_*b*_ (Ti 2p_1/2_) = 463.30 eV; (2) Ti^3+^ was from Ti_2_O_3_ species, with the binding energies locating at *E*_*b*_ (Ti 2p_3/2_) = 457.50 eV and *E*_*b*_ (Ti 2p_1/2_) = 463.30 eV [7].

### 3.3. Optical Absorption Spectra

**Figure 4:**
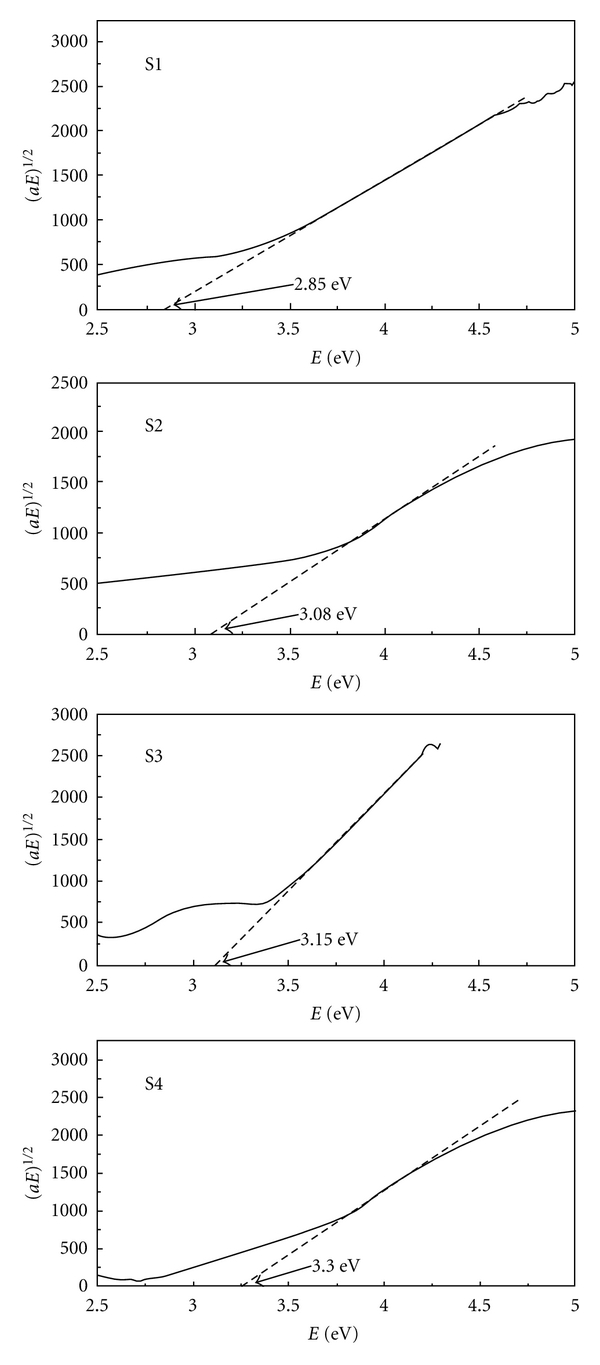
Tauc plot of (*αhγ*)^1/2^ as a function of photon energy (*hγ*) for different amount of La_2_O_3_-doped TiO_2_ thin films.

The optical properties of the films were investigated by UV-visible spectroscopy measurements in the wavelength range of 200–700 nm. Tauc relationship *αE* = *B*(*E*−*E*_*g*_)^2^ [8] was used to evaluate the optical gap values (*E*_*g*_).  Figure 4 shows the plot of (*αhγ*)^1/2^ as a function of photo energy (*hγ*) for the films. It is clearly seen that the optical gap varies significantly with the La_2_O_3_ concentration in TiO_2_ films, and *E*_*g*_ of the samples increases from 2.85 to 3.3 eV with increasing of the La_2_O_3_ content in TiO_2_ films from 0 to 3.64%. It is known that the quantum confinement will affect the electronic properties if the radius of the semiconductor particle is commensurable or smaller than the Bohr radius. As for TiO_2_, the effect of quantum-sized confinement is expected if the particles become smaller than 10 nm (usually 2-3 nm) [9, 10]. According to the measuring results of SEM (showed in Figure 2), the increase of band gap of TiO_2_ could be due to the presence of La_2_O_3_. For pure La_2_O_3_, the band gap is around 4.3 eV, higher than pure TiO_2_.

The interaction between blood and contacting biomaterials is very complicated and the detailed mechanism of hemocompatibility of TiO_2_ films is still not clear. It is demonstrated that the formation of thrombus on biomaterial is correlated with electrons transferring from the inactive state of fibrinogen to the surface of the biomaterial. During the process, fibrinogen decomposes to fibrinomonomer and fibrinopeptides. After decomposition, fibrinomonomers give rise to polymers and cross-linking and finally form an irreversible thrombus [11]. So fibrinogen plays an important role in hemostasis [12]. Not only does it participate in the coagulation cascade, but also it promotes adhesion of platelets and activates them when adsorbed onto certain solid surfaces [13]. Therefore, after the adsorption of fibrinogen, it is very important to postpone the decomposition of the protein for a biomaterial with good blood compatibility. It is related to the semiconductor property of TiO_2_ films.

It is found that the optical band gap La_2_O_3_-doped TiO_2_ film is about 3.3 eV, and the Ti^2+^ and Ti^3+^ states exist in the TiO_2_ film. This makes the film exhibit n-type semiconductor properties. It is proved that fibrinogen has an electronic structure similar to an intrinsic semiconductor with a band gap of 1.8 eV. When fibrinogen adsorbs on the surface of TiO_2_ films, the transfer of electrons is determined by the Fermi level of the film and fibrinogen. In order to inhibit the transfer of the electrons from fibrinogen to La_2_O_3_-doped TiO_2_ film, the Fermi level of La_2_O_3_-doped TiO_2_ film must be close to the bottom of the conduction band, that is to say, reducing the work function of the film. The existence of Ti^2+^ and Ti^3+^ exhibits this effect. Thus, the perfectly electronic characteristics such as wider band gap and lower work function due to La dopant make La_2_O_3_-doped TiO_2_ films exhibit better blood compatibility.

## 4. Conclusion

This study represents the relationship between the electronic structure and hemocompatibility. With the increasing of the La_2_O_3_ concentrations, the TiO_2_ films become smooth, and the grain size becomes smaller. The band gap of the samples increases from 2.85 to 3.3 eV with increasing of the La_2_O_3_ content in TiO_2_ films from 0 to 3.64%. Based on the contact angles and platelet adsorption experiments, La_2_O_3_-doped TiO_2_ films not only possess excellent surface properties of absorbing human serum albumin (HAS) preferentially, but also exhibit n-type semiconductor properties, which farther inhibit the transfer of the electrons from fibrinogen to TiO_2_ films.
